# Perturbed microRNA Expression by *Mycobacterium tuberculosis* Promotes Macrophage Polarization Leading to Pro-survival Foam Cell

**DOI:** 10.3389/fimmu.2017.00107

**Published:** 2017-02-08

**Authors:** Pankaj Kumar Ahluwalia, Rajan Kumar Pandey, Prabodh Kumar Sehajpal, Vijay Kumar Prajapati

**Affiliations:** ^1^Department of Molecular Biology and Biochemistry, Guru Nanak Dev University, Amritsar, Punjab, India; ^2^Department of Biochemistry, School of Life Sciences, Central University of Rajasthan, Ajmer, Rajasthan, India

**Keywords:** *Mycobacterium tuberculosis*, macrophage polarization, granuloma, foam cells, miRNAs

## Abstract

Tuberculosis (TB) is one of the prevalent causes of death worldwide, with 95% of these deaths occurring in developing countries, like India. The causative agent, *Mycobacterium tuberculosis* (MTb) has the tenacious ability to circumvent the host’s immune system for its own advantage. Macrophages are one of the phagocytic cells that are central to immunity against MTb. These are highly plastic cells dependent on the milieu and can showcase M1/M2 polarization. M1 macrophages are bactericidal in action, but M2 macrophages are anti-inflammatory in their immune response. This computational study is an effort to elucidate the role of miRNAs that influences the survival of MTb in the macrophage. To identify the miRNAs against critical transcription factors, we selected only conserved hits from TargetScan database. Further, validation of these miRNAs was achieved using four databases *viz*. DIANA-microT, miRDB, miRanda-mirSVR, and miRNAMap. All miRNAs were identified through a conserved seed sequence against the 3′-UTR of transcription factors. This bioinformatics study found that miR-27a and miR-27b has a putative binding site at 3′-UTR of IRF4, and miR-302c against IRF5. miR-155, miR-132, and miR-455-5p are predicted microRNAs against suppressor of cytokine signaling transcription factors. Several other microRNAs, which have an affinity for critical transcription factors, are also predicted in this study. This MTb-associated modulation of microRNAs to modify the expression of the target gene(s) plays a critical role in TB pathogenesis. Other than M1/M2 plasticity, MTb has the ability to convert macrophage into foam cells that are rich in lipids and cholesterol. We have highlighted few microRNAs which overlap between M2/foam cell continuums. miR-155, miR-33, miR-27a, and miR-27b plays a dual role in deciding macrophage polarity and its conversion to foam cells. This study shows a glimpse of microRNAs which can be modulated by MTb not only to prevent its elimination but also to promote its survival.

## Introduction

**Figure 1:**
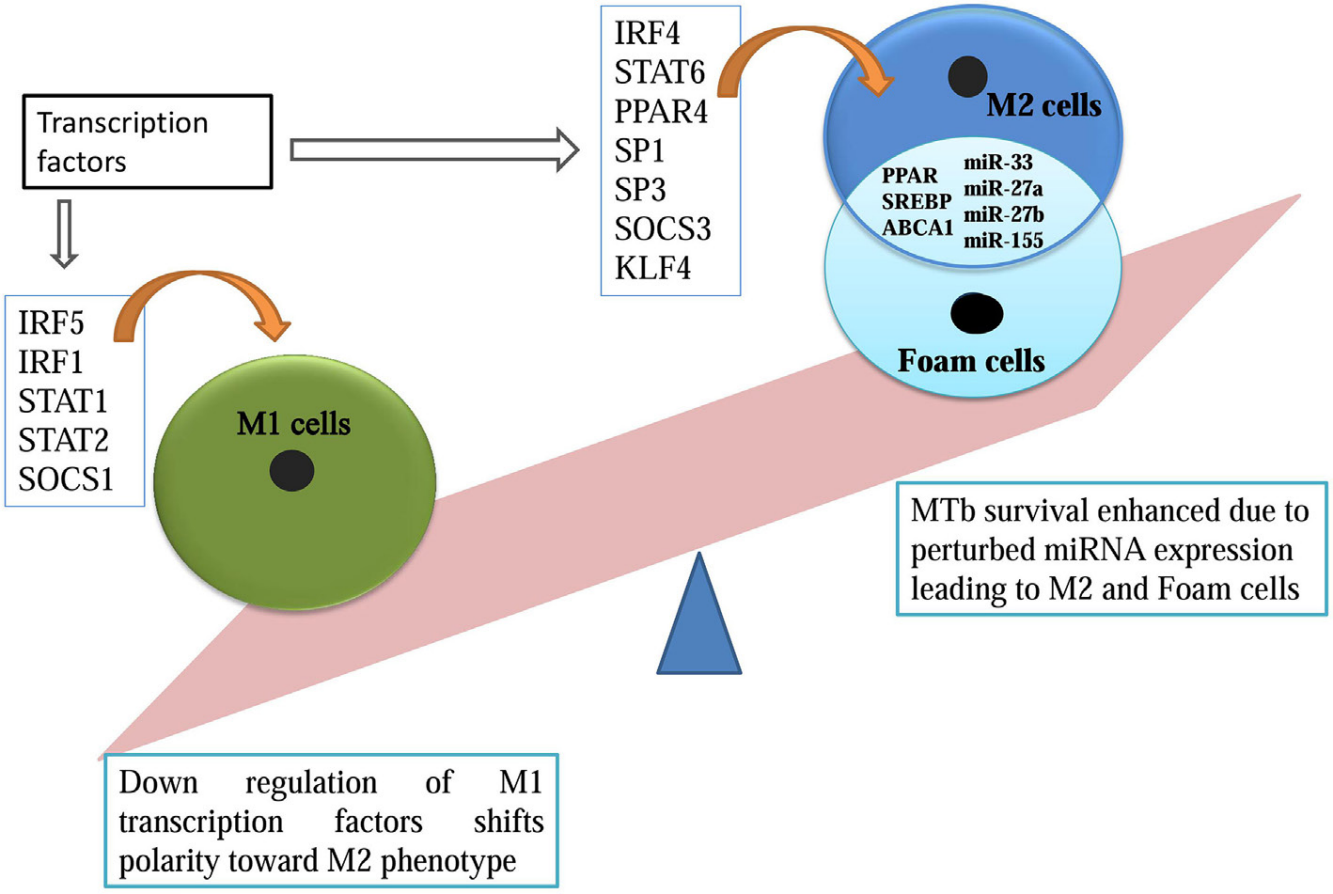
**microRNA overlapping M2 and foam cell formation promotes *Mycobacterium tuberculosis* survival**.

Tuberculosis (TB) is an infectious disease which is caused by strains of genus *Mycobacteria*. The predominant pathogenic species that infects humans is *Mycobacterium tuberculosis* (MTb). It is a pulmonary pathogen as its typical life cycle begins in lungs of human but can spread to other organs and tissues and causes extra-pulmonary TB (1). It spreads through the air when diseased person coughs or sneezes (2). In 2014, 9.6 million new cases of TB were diagnosed and TB was a cause of death for 1.5 million patients worldwide (3). One-third of the world’s populations, that is, nearly 2.5 billion individuals are infected with MTb, but remain asymptomatic; among them, only 10% will develop clinically active TB in their lifetime (1). Though TB cases are declining due to effective antibiotic regiments, interest in TB has resurfaced due to the global emergence of multiple drug-resistant (MDR) and extreme drug-resistant (XDR) strains. As new resistant strains are making antibiotics obsolete, it has become pertinent to study the host–pathogen interaction to develop strategies which could augment host to fight against the pathogen. To achieve this, understanding of the host immune dynamics at its fundamental level is essential. The evolutionary arms race has endowed both the pathogen and host with several advantages to counter each other strategies. The fight between pathogen effectors and host immunity determines whether the pathogen is eliminated, made ineffective, or causes disease. When MTb is inside the human lung, phagocytic cells act as antigen-presenting cells (APCs) to neutralize the pathogen. APCs, such as neutrophils, dendritic cells, and macrophages, are known to concomitantly act to initiate T cell-based adaptive immunity to clear out the infection (4). Macrophages are of central importance as they possess a battery of immunological processes to kill the pathogen. Macrophages normally generate toxic nitrogen and oxygen radicals and induce autophagy and apoptosis to eliminate the pathogen. These capabilities are severely compromised by MTb and can, in turn, be manipulated for its survival. MTb has been experimentally shown to evade both apoptosis and autophagy by altering the intracellular machinery of the macrophage (5). In macrophage, a pathogen is normally phagocytosed and gets encased in endosome inside the cytosol. When this endosome matures, it fuses with the lysosome whereby the pathogen is neutralized, but MTb has the capacity to perturb the intracellular trafficking. It incapacitates phagosomal acidification by arresting its fusion with the lysosome (6). This crucial hindrance, which prevents phagolysosome maturation, leads to the survival of MTb inside the macrophage. This feat is achieved by MTb due to its interference with several intracellular pathways. It inhibits Rab5-dependent endosome maturation, modulates Ca^2+^ and PI3P-based signaling along with the modification of the actin cytoskeleton to prevent its fusion with the lysosome (7). By modulating intracellular mediators, MTb establishes an immunological block that leads to a chronic inflammatory condition in which it continues to persist. Host counteracts the onslaught and initiates an adaptive immune response by forming a granuloma, an immunological barricade to contain the further infection. By the time infection progresses, T-helper (T_H_1) cell with its cytokine secretions, such as tumor necrosis factor-alpha (TNF-α) and interferon-gamma (IFN-γ), propagates the adaptive limb of the immunity (8). The granuloma is established when a sufficient inflammatory response is generated by T_H_1 cells along with the expression of inflammatory chemokine (9). The main effector cells which are affected by cytokines of T_H_1 cells are macrophages. T_H_1 response primes macrophages by upregulating major histocompatibility complex (MHC)-2 and activating appropriate signaling pathways necessary for the elimination of intracellular pathogens. MTb has the capacity to resist the deadly assault of the macrophage. Other than that, macrophages show a high level of plasticity and can differentiate into two opposite phenotypes (10). M1 macrophages are classically activated macrophages, which are immune-stimulatory and are influenced by cytokines, such as IFN-γ and TNF-α. M2 cells are alternatively activated macrophages, which are characterized by their immuno-suppressive properties, such as enhanced IL4, IL10, and IL-13 response (11). There is a lot of ambiguity in M1/M2 nomenclature, but certain transcription factors and cytokines have been shown to differentiate these extreme phenotypes (Figure 1).

Apart from M1/M2 plasticity, macrophages can also transform into foam cells, which are rich in lipid and cholesterol (12). Foamy macrophages are cells with deregulated lipid metabolism (13, 14). The homeostatic balance between the influx and efflux of low-density lipoprotein (LDL) comprising cholesterol, triacylglycerides, and phospholipids, is compromised in these cells. This modification is achieved in two steps, first, scavenger receptors and CD36, leads to increased uptake of LDLs by the macrophages. Second, transporters like ABCA1 that regulate the efflux of excess cholesterol are affected inside these macrophages. ABCA1 knockdown has been associated with exacerbated conditions of foam cells. Increased uptake of LDLs and later its compromised efflux suits the nutritional requirement of MTb (13). MTb has also been shown to modulate the ketone body synthesis to accumulate lipid bodies inside the macrophages (14). Peroxisome proliferator-activated receptor (PPAR) group of transcription factors have also been implicated in both M2 transition and later in foam cell formation. PPARs upregulate metabolism genes and also represses M1 phenotypic marker (15). All of these factors are susceptible to modulation by MTb for its survival. These modified macrophages in the form of foamy cells are shown to be a predominant niche for MTb in granuloma (16) (Figure 1).

In TB, granuloma formation is an immunologically dynamic process which involves both innate and adaptive mechanisms. Few studies have focused on M1 and M2 macrophages bias in granuloma (17). The confirmed presence of M1, M2, and foamy macrophages in granuloma warrants further investigations to understand its transition and its role in the pathology of the disease. We have suspected the role of microRNAs against some transcription factors which transcribe critical genes that affect the M1/M2/Foamy macrophage continuum. miRNAs are evolutionarily conserved, 20–24 nucleotide small non-coding RNA, which inhibits the expression of genes by binding to the 3′-UTR of the transcribed mRNAs. The recognition of mRNA nucleotides by 6–8 nucleotides long seed region of miRNA is sufficient to inhibit the gene expression (18). The miRNA–mRNA complex leads to mRNA destabilization, translational inhibition, or mRNA degradation. miRNA has been shown to play diverse roles from affecting developmental pathways to immunological mechanisms (19). The specific variations in miRNA expression during immune cell development are increasingly being appreciated. Its aberrant expression has been linked to infections, aging, and other pathological outcomes (20). As monocyte transition to macrophage is a continuous process, the perturbed expression of miRNA can influence its phenotype under certain temporal and spatial conditions. In this study, we have identified transcription factors which can regulate macrophage polarization during MTb infection. In fact, several studies have corroborated that levels of transcription factors present in cytosol influence the gene expression profile of a particular cell (21, 22). MTb-associated miRNA modulation can lead to a quantitative variation of transcription factors inside the macrophage either by directly altering the transcription factor or by influencing other proteins interacting with the transcription factors. There has been evidence that PtpA secreted by MTb directly dephosphorylates p-Jnk and p-p38, thus modulating cytokine expression (23).

In this computational study, we have identified key miRNAs that can be upregulated or repressed to skew the macrophage polarity to pro-survival M2 phenotype. Transcription factors like IRF5, IRF1, SOCS1, STAT1, and STAT2 promotes inflammatory M1 macrophage, and its MTb-based suppression enhances its survival. Transcription factors expressed by M2 macrophages, such as IRF4, SP1, SP3, STAT6, SOCS 2, SOCS 3, PPAR, and KLF6, were also analyzed in this study. Their upregulation boosts the survival of MTb (Figure 1). This study revealed novel MTb-associated miRNAs which bind to the 3′-UTR of the aforesaid transcription factors. The variation in the miRNA profile decides the fate of the macrophage to which survival of MTb is intertwined. Later, we analyzed few microRNAs which fulfills the dual role of transition from M2 phenotype to nutritional rich foam cells. Similar computational approaches have been used in deciphering the role of miRNAs in other human disorders (24, 25).

## Materials and Methodology

### Identification of Target Transcription Factors

MTb survival is greatly enhanced when macrophage polarity is shifted toward M2 macrophage. We have identified key transcriptional factors from the published literature that were experimentally shown to affect macrophage polarization state and are listed in Table 1.

**Table 1:** **Transcription factors and their functional aspects toward the plasticity of M1 or M2 phenotype**.

S. No.	Type of phenotype	Transcription factors	Full name	Function	Reference
1	M1 phenotype	IRF5	Interferon regulatory factor 5	Required for MyD88 signaling and drives pro inflammatory response	(26)
2		IRF1	Interferon regulatory factor 1	Interacts with Myd88 and activates pro-inflammatory genes	(27)
3		SOCS 1	Suppressor of cytokine signaling 1	High SOCS 1/SOCS 3 ratio drives toward M1	(28)
4		STAT1	Signal transducer and activator of transcription 1	Required for interferon-gamma-based pro-inflammatory genes activation	(29)
5		STAT2	Signal transducer and activator of transcription 1	Forms heterodimer with STAT1	(29)

6	M2 phenotype	IRF4	Interferon regulatory factor 4	Promotes M2	(29)
7		SP1	Specificity protein 1	Transcription factor for IL-10 gene	(30)
8		SP3	Specificity protein 3	Transcription factor for IL-10 gene	(30)
9		PPAR-δ	Peroxisome proliferator-activated receptor delta	Promotes anti-inflammatory M2 phenotype	(31)
10		PPAR-α	Peroxisome proliferator-activated receptor alpha	Promotes M2	(32)
11		PPAR-γ	Peroxisome proliferator-activated receptor gamma	Leads in fatty acid metabolism and sustains M2 phenotype	(15)
12		SOCS2	Suppressor of cytokine signaling 2	Highs SOCS 2 leads to M2	(28)
13		SOCS 3	Suppressor of cytokine signaling 3	High SOCS 3/SOCS 1 ratio drives toward M2	(28)
14		STAT6	Signal transducer and activator of transcription 6	Induces M2 specific genes	(29)
15		KLF4	Kruppel-like factor 4	Inhibits M1 promotes M2	(33)

### Resources Used for miRNA Prediction

We have used five freely accessible databases which are available online to predict potential miRNAs against target genes (Table 2). For this study, first, we submitted target gene ID into TargetScan database to identify matching conserved miRNA sequences. TargetScan returned queries in two sets, first set, conserved sequences with a high probability of binding between seed region of mRNA and second set comprised of poorly conserved miRNA. The algorithm threshold was set automatically according to the length of the seed sequence. In all the resulting hits of the miRNAs, the length of the seed sequence matching the mature mRNA was ≥7mers. The context ++ percentile score was used to curate results on the basis of a statistical model to predict miRNA/mRNA interaction (34). Context ++ percentile score ranks microRNAs according to its efficacy of binding. Only conserved hits (with context ++ score ≥90%) were filtered and authenticated in other databases. Other four web servers used in this study were: DIANA-microT, which identifies miRNA recognition elements by comparing given miRNA with mock miRNA sequence (35), miRDB utilizes support vector machines for target prediction (36), miRanda-mirSVR is an online miRNA analysis tool which utilizes a new machine learning method which is trained on *in vitro* miRNA transfection experiments on HeLa cells (37), and miRNAMap primarily considers Gibbs free energy and miRanda score to predict target sites (38).

**Table 2:** **List of online resources used for microRNA prediction**.

S. No.	Tool name	Uniform resource locator	Reference
1	TargetScan	http://www.TargetScan.org	(34)
2	miRDB	http://miRDB.org/miRDB	(39)
3	miRANDA-mirSVR	http://www.microrna.org	(40)
4	DIANA-microT	http://diana.imis.athena-innovation.gr/DianaTools/index.php?r=microT_CDS/index	(35)
5	miRNAMap	http://mirnamap.mbc.nctu.edu.tw	(38)

## Results

### The MTb-Dependent Downregulation of M1 Phenotypic Properties

Macrophage polarization leads to two states, one is a pro-inflammatory M1 state, which encompasses IFN-γ-mediated response and microbicidal action. The second state is an M2 state, which is an anti-inflammatory state pre-dominated by IL4- and IL10-mediated cell profile. MTb-dependent perturbations in the host’s microRNA profile would enhance its survival only if it could negate the host’s lethal response. To identify microRNAs against transcription factors required for M1 cell, we submitted IRF5 (GENE ID 3663) for miRNA search in TargetScan database. The data search revealed that miR-302c and miR-520b were conserved with the high context ++ score. But only miR-302c, which targets 3′-UTR position at 452–459 of IRF5, was validated on miRanda-mirSVR and miRDB databases (Table 3).

**Table 3:** **Predicted microRNA against different transcription factors playing role in M1/M2 plastcity**.

S. No.	Transcription factors	TargetScan	miRanda–mirSVR	miRDB	microT	miRNAMap
1	IRF5	miR-302c miR-520b	miR-302c miR-520b	miR-302c –	– –	– –

2	IRF1	miR-454 miR-130a miR-130b	miR-454 miR-130a miR-130b	miR-454 miR-130a miR-130b	miR-454 miR-130a miR-130b	– – –

3	SOCS 1	miR-155 –	miR-155 miR-7c	– miR-7c	miR-155 –	miR-155 –

4	STAT1	–	–	miR-1252-5p	miR-1252	–

5	STAT2	miR-3202	–	miR-3202	miR-3202	–

6	IRF4	miR-27a miR-27b	miR-27a miR-27b	miR-27a miR-27b	miR-27a miR-27b	– –

7	SP1	miR-135a-5p miR-135b-5p	miR-135a-5p miR-135b-5p	miR-135a-5p miR-135b-5p	miR-135a-5p –	– –

8	SP3	miR-129-5p	miR-129-5p	miR-129-5p	–	–

9	SOCS2	miR-132 miR-212	– –	– –	miR-132 miR-212	miR-132 miR-212

10	SOCS 3	miR-455-5p	miR-455-5p	miR-455-5p	miR-455	–

11	KLF4	miR-34c miR-449	miR-34c miR-449	miR-34c miR-449	miR-449 –	miR-34c miR-449

12	STAT6	miR-135a-5p miR-135b-5p	miR-135a miR-135b	miR-135a-5p miR-135b-5p	– –	– –

13	Peroxisome proliferator-activated receptor (PPAR)-δ	miR-138-5p	miR-138	miR-138-5p	miR-138-5p	–

14	PPAR-γ	miR-454 miR-301a	miR-454 miR-301a	miR-454 miR-301a	– –	miR-454 –

15	PPAR-α	miR-19a miR-19b	– –	miR-19a miR-19b	– –	miR-19a miR-19b

IRF1 has been associated with M1 macrophages. IRF1 interacts with Myd88 and activates inflammatory genes. miR-454, miR-130a, and mir-130b have binding affinity for 3′-UTR position at 394–401 of IRF1. Its downregulation will prevent M1-specific response (Table 3).

The STAT family of transcription factors plays a differential role in the outcome of macrophage. miR-1252 and miR-3202 showed complementarity for STAT1 and STAT2, respectively in ≥2 databases. Downregulation of these transcription factors will lead to a reduced inflammatory response (Table 3).

**Figure 2:**
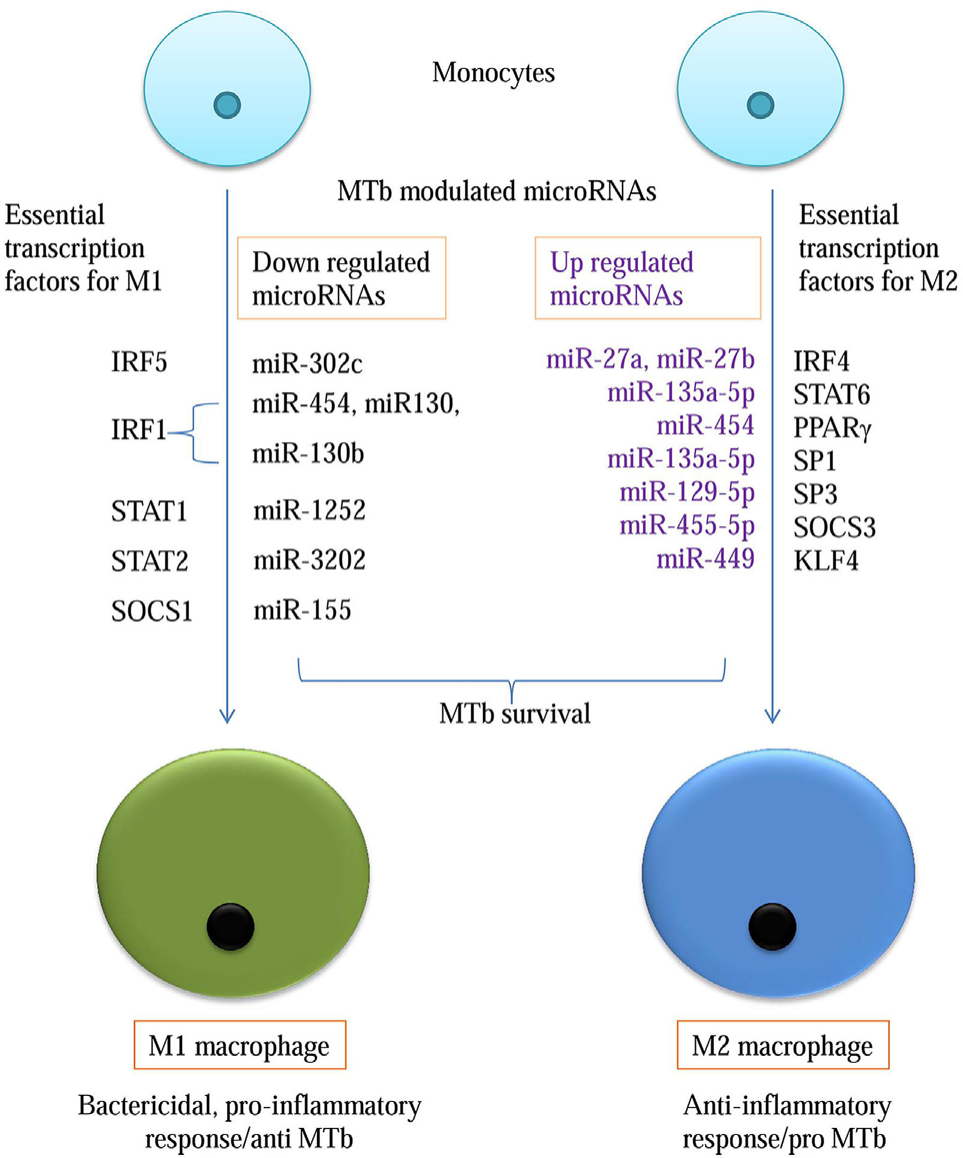
**microRNA promoting M2 phenotype for *Mycobacterium tuberculosis* survival**.

Suppressor of cytokine signaling (SOCS) affects signaling cascade influencing M1 and M2 outcome. SOCS 3 is an essential transcription factor for M1 macrophage conversion. The miR-455-5p was found to possess an affinity for position 1564–1570 of 3′-UTR of SOCS3. The miR-455-5p target site was confirmed in four databases except for miRNAMap. The expression of miR-455-3p will downregulate SOCS 3 expression which is essential for M1 phenotype, thus promoting M2 phenotype (Table 3; Figure 2).

### MTb-Dependent Macrophage Imbalance toward M2 Phenotype

IRF4 is an essential transcription factor for M2 phenotype generation but is dispensable for M1 development. Two microRNAs, miR-27a and miR-27b, showed conserved binding to position 437–444 of IRF4 3′-UTR with high P_CT_ (Probability of Conservation) and context ++ percentile score. The binding of these miRNAs was corroborated on miRanda-mirSVR, miRDB, and microT databases. Among the STAT family of transducers, STAT6 has been positively associated with an M2 cell type. TargetScan, miRDB, and miRanda-mirSVR revealed putative binding sites for miR-135a-5p and miR-135b-5p at position 1100–1107 of STAT6. These microRNA when upregulated, would promote M2 phenotype (Table 3).

High SOCS1 to SOCS3 ratio affects signaling cascade differentially by promoting M2 macrophage. When submitted in TargetScan, miR-155-5p binds at position 24–31 of SOCS1 3′-UTR. Its affinity was confirmed on miRanda-mirSVR, microT, and microRNAMap. Conserved microRNAs predicted on TargetScan for SOCS2 3′-UTR at position 1535–1542 was miR-132 and miR-212. Its affinity was simultaneously confirmed through TargetScan, microT, and miRNAMap databases (Table 3).

SP1 and SP3 are known to transcriptionally upregulate IL-10 gene. TargetScan output has shown that SP1 has a conserved target at 344–350 nucleotide position of 3′-UTR for miR-135a-5p and 135b. The validation of miR-135a-5p was confirmed in all the four databases except miRNAMap. For SP2, miR-129-5p at 93-99 position of 3′-UTR was confirmed in TargetScan, miRanda-mirSVR, and miRDB. Downregulation of microRNAs against SP1 and SP2 leads to upregulation of IL10 expression, in turn, promoting M2 phenotype (Table 3).

High activity of KLF4 is associated with M2 macrophages but is significantly reduced in M1. Its 3′-UTR sequence at 25–32 nucleotides shows conserved targets for miR-449b-5p and miR-34c-5p (Table 3; Figure 2).

microRNA have been also implicated in lipid metabolism and can potentially assist in the transition of M2 cell to foamy macrophage. Our data-mining in multiple databases confirmed that miR-155 has the conserved binding site against ABCA1. miR-33 which play a role in lipid deregulation binds to the 3′-UTR of NOD2. Another critical microRNA, miR-27a/b has a putative binding site at 3′-UTR of IRF4.

Among all the 15 transcription factors analyzed, 1 had hit in all five databases, 7 microRNAs were positively authenticated on four databases and 6 had hits in three databases. Except for one gene all the microRNAs were validated in ≥3 databases with different algorithms.

## Discussion

Tuberculosis involves combined effort of innate and adaptive immunity to clear out the infection. Macrophages are the central immune cells, as they possess an arsenal of toxic mechanisms sufficient to kill the pathogen. MTb-dependent modulation of macrophage, whereby it not only survives but also thrives, is one of the great evolutionary adaptations. In the host, macrophages’ plasticity yields predominantly pro-inflammatory M1 macrophages and anti-inflammatory M2 macrophages. For MTb, the advantage of M2 cells could be gauged by the fact that it downregulates the antigen presentation molecules and co-stimulatory receptors, in turn impairing the activation of T_H_1 cells (41). The absence of T_H_1 response coupled with immune manipulation to generate M2 creates a comfortable niche for this pathogen. Add to it, the capacity to transform macrophage into a foam cell, MTb successfully manipulates the host to generate a safe harbor for its long-term survival (16). The crucial molecular factors which are essential for deciding the phenotypic outcome of a macrophage is of great interest as the survival of pathogens is dictated by the lethality of macrophage response. As infection by MTb progresses, the defensive action of host creates an immunological barricade called a granuloma. There has been evidence suggesting the presence of both types of macrophages in the granuloma (17). During the initial stages of infection, M1 type of macrophages predominates, but loses out to M2 as the infection progresses (17). M1/M2 polarization is based on the differential expression of genes in a macrophage. For example, IL-23- and IL-12-secreting M1 macrophages promote mycobacterial killing, but IL-10-secreting M2 expression subverts its killing (41). Expression of these genes is controlled by specific transcription factors, which express a single gene or a set of genes (42). The fine-tuning of gene expression with respect to transcription factors could be achieved in two ways. First, the downregulation or upregulation of the transcription factors in the cell leads to the corresponding effect on the quantity of the gene product. Second, modification of transcription factor also influences the gene expression. Certain transcription factor such as NFAT must be dephosphorylated before they can translocate to the nucleus (43). Some studies have shown that the quantity of transcription factor as a key variable in deciding the inflammatory status of the macrophage (21, 22). We have pointed out that MTb-dependent miRNAs modulation of transcription factors might be one of the molecular factors that have the capacity to tip the balance toward pro-survival M2 phenotype.

In this computational study, we have uncovered few critical transcription factors which influence the M1/M2 plasticity and are potential targets modulated by MTb. Indeed, many other studies are also pointing at the role of microRNAs in various immune cells, including macrophages (20). Experimentally several studies have documented MTb-dependent miRNA modulation (44, 45). The pathogen downregulates inflammatory molecules IFN-γ, TNF-α, and IRAK1 by modulating several miRNAs (46–48). In this study, by analyzing multiple databases, we have filtered critical micro RNAs that can affect the availability of TFs. Initial hits were selected from TargetScan as it uses P_CT_ and context ++ percentile score. Higher score translates to a better probability of binding with the given mRNA. These filtered miRNAs were further analyzed by other databases.

The interferon regulatory elements (IRF) are transcription factors which upregulate the transcription of a specific set of genes and play a significant role in deciding the outcome of macrophage phenotype. For M1 macrophage, IRF5 is the critical transcription factor which influences the inflammatory response of the macrophage (26). IRF5 depended production of IL-12 by M1 macrophage is required to IFN-γ-dependent pathway in T_H_1 cells. IFN-γ then potentiates the macrophages to unleash its antibacterial arsenal (49). IRF5 also inhibits the transcription of IL10, thus negatively regulates M2 phenotype. Its upregulation is synonymous with M1 and down regulation leads to M2 cells. IRF1 also plays its part by inducing Myd88-dependent pathway, but recent evidence has also suggested its association with BATF1 which leads to the expression of several host protective genes against MTb (50). Its knockdown lead to severe downregulation of IFN-γ-dependent pro-inflammatory limb (50). IRF5 and IRF1 down-modulation by miRNAs prolong the survival of MTb in macrophages.

IRF4, another transcription factor is essential for generating M2 phenotype. IRF 4 is known to associate with Jmjd3, an H3K27 demethylase, and has been implicated in the promotion of M2 phenotype (51). We found out that miR-302c putatively controls IRF5 expression, and miR-27a and miR-27b regulate IRF4 expression. For the promotion of M2 macrophage, it is essential to downregulate IRF5 by upregulating miR-302c. Expression of IRF4 can be affected by downregulating miR-27a and miR-27b. When perturbed by MTb, these miRNA can significantly alter the macrophage polarization status, thus providing a means to MTb for its survival.

STATs are an intracellular transducer that regulates the expression of several immune genes in macrophages. These participate by expressing higher MHC-2, IL-12, and Nitric oxide synthase (NOS), necessary for killing of the pathogen (52). STAT1 and STAT2, transducers play their part in M1 polarization, they form a heterodimer to induce transcription of several microbicidal genes. miR-1252 and miR-3202 against STAT1 and STAT2 will downregulate M1-specific response.

Suppressor of cytokine signaling proteins has been shown to play a key role in M1 and M2 macrophage polarization. Expression of SOCS is highly context dependent and is actively triggered once the macrophage is activated. SOCS plays a dampening effect on JAK/STAT signaling pathways through its association with phosphorylated tyrosine. SOCS also utilizes proteasome machinery to degrade signaling molecules. High SOCS1 and SOCS2 have been associated with M2 polarization, and high SOCS3 promotes M1 phenotype (53, 54). Downregulation of miR-155-5p against SOCS1 will result in corresponding increase in SOCS1. The down-modulation of miR-132 will increase SOCS2. High SOCS1 and SOCS2 will promote pro-survival M2 phenotype. At the same time, MTb has the capacity to increase miR-455 expression which possesses conserved complementarity against SOCS3 and thus, prevents the cell from generating M1-specific response.

As IFN-γ and TNF-α are associated with M1 macrophages, IL-4 and IL-10 are known to be associated with M2 macrophages (10). SP1 and STAT6 both play an important role in the outcome of the M2 phenotype. STAT6 plays its part in IL-4-dependent cell signaling (55). SP1 has been shown to bind to IL10 motif, thus regulating its expression which is critical for M2 cell (30). IL-10 promotes anti-inflammatory genes and is associated with a dampening of the immune response. Our computation study predicted the affinity of miR-135a and miR-135b to these mRNA. MTb-dependent downregulation of this microRNA will be advantageous to MTb as it greatly enhances the formation of M2 phenotype.

KLF4 is also essential for M2 as it is highly expressed in M2 macrophages but is significantly reduced in M1 macrophages (33). It binds to STAT6 and sequesters co-activators required for NF-κB. As it is an important transcription factor which expresses an inflammatory gene, its unavailability shifts the macrophage toward M2 path. Our study found that miR-34c and miR-449 have conserved targets against KLF4. MTb-dependent upregulation of KLF4 through negative modulation by these microRNAs might lead to M2 phenotype.

### Modulation of Transcription Factors for Foam Cells in the Granuloma

Foam cells are lipid-rich macrophages and are abundantly present in the granuloma (12). These are nutritionally rich cells where phagocytic and bactericidal activities are permanently compromised (16). MTb has remarkably long survival time in foam cells. It is a nutritionally rich microenvironment for MTb. In this safe haven, MTb can enter dormancy and can actively reproduce when suitable conditions emerge (56). Molecular factors affecting foam cell formation are of critical interest. PPARs are nuclear receptors, which act as transcription factors for genes playing an essential role in cellular metabolism, predominantly, glucose and lipid metabolism. Apart from this function, PPAR-α, -δ, -γ has shown the capacity to influence macrophage plasticity. They actively promote M2 phenotype by repressing inflammatory genes through interference with the pro-inflammatory transcription factors, such as NF-κB (15). Development of human monocyte is skewed toward anti-inflammatory M2 when in presence of PPAR-γ. PPAR-γ activation correlates positively with M2 phenotypic markers. In one step ahead, PPAR-α and PPAR-γ have been implicated in ABCA1-mediated cholesterol efflux from macrophages (57). Thus, PPAR-mediated pathways play a role both in M2 macrophage generation and foam cell formation by regulating cholesterol efflux. MTb has been shown to depend on cholesterol metabolism for its long-term persistence (58). Our data mining in multiple databases confirmed that miR-155 has the conserved binding site against ABCA1. By upregulating miR-155 late in infection, MTb has the capacity to block cholesterol efflux, by downregulating ABCA1, thus, securing its cholesterol requirements inside the foam cells. Experimental evidence has also suggested that miR-155 acts by directly repressing HBP1 expression, in turn, promoting lipid uptake (59). Interestingly, in TB patients, studies have shown increased expression of miR-155 (60, 61). By expressing miR-155, MTb can negate microbicidal cytokines and on the same hand prime foam cell formation. There is also a report which shows that mir-155 enhances autophagy (23) but autophagy itself is modulated in the presence of anti-inflammatory cytokines (62). miR-155 play role in suppressing inflammation and later lipid uptake creates ideal conditions for its survival and abrogates autophagy.

One of the transcription factors related to lipid metabolism is SREBP. It is required for upregulation of fatty acid and cholesterol metabolism genes. SREBP1 and SREBP2 also express miR-33 which is present in its intronic region. miR-33 binds to ABCA1 and prevents the efflux of cholesterol from the cell (63, 64). Increased SREBP expression will not only extend lipolytic machinery but, through miR-33 expression, can also block cholesterol efflux, thus promoting foam cell formation. The modulation of miR-33 will again serve dual purposes for MTb by increasing fatty acid metabolism genes and at the same time reducing the outflow of cholesterol. Cholesterol and fatty acids are known to be nutrients for MTb, and its accumulation enhances its survival. Our database search also found that miR-33 binds to 3′-UTR of NOD2. Downregulation of NOD2 dampens the inflammatory response (65). In fact, a recent study found out that MTb induced expression of miR-33 lead to deregulated autophagy, a protection strategy for the MTb. It also enhanced lipid catabolism which is essential for its nourishment (66). miR-27a/b has been experimentally shown to regulate cholesterol homeostasis in THP1-macrophages (67). Our analysis has revealed that miR-27a/b has a putative binding site at 3′-UTR of IRF4. Pathogen-mediated modulation of this microRNA will subvert the immune response along with metabolic perturbations required for its survival. Further experiments are required to fill the gaps in our current understanding of the microRNAs role in M1/M2 polarization and subsequently foam cell formation in granulomatous conditions.

Computational investigations have been conducted in cancer biology to identify critical microRNAs and their role in disease pathogenesis (25). The criteria followed in these studies are variable and at times is analyzed using a single database. In this study, the computational approach followed is highly stringent and requires the hit to be identified and validated by ≥3 databases. The hits in multiple databases are important as various databases use different algorithms and selection criterion to find microRNA/mRNA match, thus, strengthening its probability of function *in vivo*. Using this approach, we have shown that multiple microRNAs can be modulated by MTb for its survival (Table 3). MTb has adapted to manipulate host metabolism and immune response at the same time by modulating its overlapping transcription factors. Experimental evidence must corroborate the role of microRNAs that are found in this *in silico* study. Further investigations should decipher novel miRNAs that influences TB pathogenesis. Future studies might document the use of microRNAs as novel therapeutic targets for curbing the spread of TB.

## Author Contributions

PA, RP, PS, and VP conceived and designed the experiments, contributed reagents/materials/analysis tools, and wrote the paper. PA and RP performed the experiments. PA, RP, and VP analyzed the data.

## Conflict of Interest Statement

The authors declare that the research was conducted in the absence of any commercial or financial relationships that could be construed as a potential conflict of interest.
